# Research Trends and Insights in Chronic Nonspecific Low Back Pain: Bibliometric Analysis (1996–2025)

**DOI:** 10.1155/prm/2209548

**Published:** 2026-07-24

**Authors:** Wei Yan, Lingjun Kong, Tianxiang He, Xin Zhou, Guangxin Guo, Qingguang Zhu, Xiaobing Xi, Min Fang

**Affiliations:** ^1^ Department of Clinical Medicine School, Shuguang Hospital, Shanghai University of Traditional Chinese Medicine, Shanghai 201203, China, shutcm.edu.cn; ^2^ Department of Orthopaedics, Ruijin Hospital, Shanghai Jiao Tong University School of Medicine, Shanghai 200025, China, shsmu.edu.cn; ^3^ Department of Tuina, Shuguang Hospital, Shanghai University of Traditional Chinese Medicine, Shanghai 201203, China, shutcm.edu.cn; ^4^ School of Acupuncture-Moxibustion and Tuina, Shanghai University of Traditional Chinese Medicine, Shanghai 201203, China, shutcm.edu.cn; ^5^ Yue Yang Hospital of Integrated Traditional Chinese and Western Medicine, Shanghai University of Traditional Chinese Medicine, Shanghai 200437, China, shutcm.edu.cn; ^6^ Institute of Tuina, Shanghai Institute of Traditional Chinese Medicine, Shanghai 200437, China

**Keywords:** bibliometric analysis, chronic nonspecific low back pain, CiteSpace, VOSviewer

## Abstract

**Background:**

Chronic nonspecific low back pain (CNLBP) is a major global driver of disability, with increasing attention toward individualized, evidence‐based, and function‐oriented interventions. To delineate the global CNLBP research landscape, we conducted a bibliometric and visualization analysis covering the period from 1996 to 2025.

**Methods:**

Publications were retrieved from the Web of Science Core Collection, and VOSviewer, CiteSpace, and the R package bibliometrix were used to examine publication growth, author output, contributions by countries and institutions, journal influence, keyword co‐occurrence patterns, and citation burst signals.

**Results:**

A total of 937 peer‐reviewed articles were identified, authored by 4,336 individuals across 288 journals and 65 countries. The largest share of publications was contributed by the United States, while Australia demonstrated strong international collaboration and citation impact. Institutions such as Vrije Universiteit Amsterdam, University of Southern Denmark, and University of Sydney played leading roles in CNLBP research output. Influential authors included Peter O’Sullivan and Kieran O’Sullivan, noted for contributions to psychosocial modeling and individualized rehabilitation. Keyword co‐occurrence analysis identified four thematic clusters: (1) disease definition and epidemiology; (2) evidence‐based management and health systems; (3) neuromechanical rehabilitation strategies; and (4) psychosocial dimensions and patient‐centered outcomes. Burst keyword analysis revealed recent focus on “motor control” (2021–2024), “Tampa scale” (2023–2025), and “quality of life” (2023–2025), reflecting the shift toward precision rehabilitation and multidimensional assessment.

**Conclusions:**

A comprehensive overview of the evolving CNLBP research landscape was provided through this bibliometric analysis. Findings underscore the transition from diagnostic exploration to integrated clinical interventions, with growing emphasis on function, psychological well‐being, and personalized care pathways.

## 1. Introduction

Chronic nonspecific low back pain (CNLBP) is highly prevalent and contributes substantially to global disability, with marked consequences for quality of life [1]. Globally, low back pain has consistently ranked among the leading causes of years lived with disability, underscoring its burden on both individuals and healthcare systems [2, 3]. CNLBP affects millions of people worldwide, with prevalence rates rising steadily due to aging populations, sedentary lifestyles, and increasing rates of obesity [4]. This condition can lead to chronic pain, physical limitations, and psychological distress, ultimately diminishing individuals’ ability to participate fully in both personal and professional activities [5].

Despite the significant advances in imaging, diagnostic tools, and therapeutic interventions, managing CNLBP remains a complex challenge [6]. CNLBP is often characterized by an absence of clear pathological or anatomical causes, which complicates diagnosis and treatment [7]. Current management options, including analgesic medications, physical therapy, and surgical approaches, are frequently associated with only short‐term benefit and may be ineffective for a substantial proportion of patients [8, 9]. The variability in patient response, combined with the high recurrence rate of symptoms, emphasizes the need for more personalized, long‐term approaches to management [10].

One of the core challenges in the treatment of CNLBP lies in bridging the gap between clinical practice and evidence‐based guidelines [10]. While numerous studies advocate for promoting physical activity, functional rehabilitation, and biopsychosocial interventions as effective approaches, these strategies are often underutilized in clinical settings [11]. Consequently, patients frequently face prolonged disability, reduced work productivity, and diminished quality of life. The complex nature of CNLBP, combined with the rising economic and societal costs, highlights the urgent need for better understanding and innovation in treatment approaches [12].

Given the complexity and growing prevalence of CNLBP, a comprehensive examination of the research landscape is essential to identify trends, advancements, and knowledge gaps. Bibliometrics is an interdisciplinary approach that applies mathematical and statistical methods to quantitatively and qualitatively evaluate scholarly literature [13]. Through multidimensional profiling of publication outputs, contributions from countries, institutions, journals, and authors can be characterized, and influential studies and key advances can be identified [14]. Most recently, Zang and Yan performed a large‐scale bibliometric analysis of 4,896 publications on exercise interventions for nonspecific low back pain published between 2018 and 2023, revealing emerging hotspots such as rehabilitation medicine, patient experiences, and brain‐related mechanisms [15]. When applied to CNLBP, bibliometric methods can be used to characterize publication growth, research influence, and shifts in thematic focus over time, thereby highlighting knowledge gaps and priorities for future work. Accordingly, a bibliometric analysis of CNLBP‐related literature was conducted to delineate research trends, identify influential publications and authors, and highlight emerging themes.

## 2. Materials and Methods

### 2.1. Search Strategies and Data Collection

A literature search for CNLBP was conducted in the Web of Science Core Collection (WoSCC) covering January 1, 1996, through June 16, 2025. The search strategy were (((TS=((“non‐specific chronic low back pain” OR “chronic non‐specific low back pain” OR “persistent low back pain” OR “mechanical low back pain” OR “chronic idiopathic low back pain” OR “non‐specific chronic lumbar pain”))) OR AB=((“non‐specific chronic low back pain” OR “chronic non‐specific low back pain” OR “persistent low back pain” OR “mechanical low back pain” OR “chronic idiopathic low back pain” OR “non‐specific chronic lumbar pain”))) OR TI=((“non‐specific chronic low back pain” OR “chronic non‐specific low back pain” OR “persistent low back pain” OR “mechanical low back pain” OR “chronic idiopathic low back pain” OR “non‐specific chronic lumbar pain”))) OR AK=((“non‐specific chronic low back pain” OR “chronic non‐specific low back pain” OR “persistent low back pain” OR “mechanical low back pain” OR “chronic idiopathic low back pain” OR “non‐specific chronic lumbar pain”)). The inclusion criteria were limited to English‐language publications to maintain linguistic uniformity, with only peer‐reviewed articles considered for the study. To ensure consistency in retrieval, all searches were performed on June 16, 2025.

### 2.2. Statistical Analysis

A comprehensive bibliometric analysis was conducted using multiple analytical and visualization tools. The “bibliometrix” package (R Version 4.3.3), accessible through the online platform Bibliometric Analysis, was employed to export descriptive analysis results [16]. Publication counts, average citations per publication, and country, institution, journal, and author metrics were extracted. The Hirsch index (H‐index) and impact factor (IF) were obtained from the most recent Journal Citation Reports (JCR).

VOSviewer (Version 1.6.20) was used to visualize collaboration patterns among institutions and authors and to map coauthorship, citation, and cocitation networks. Keyword co‐occurrence analysis was performed in CiteSpace (Version 6.1.R3) with time slicing set from January 1996 to July 2025, using keywords as nodes, a threshold of the top five keywords per year, and pathfinder pruning. A keyword timeline map was generated to summarize temporal changes in the CNLBP research field, with node size indicating publication frequency, link thickness indicating connection strength, and node color indicating clusters or time periods. The H‐index was used to quantify the academic impact of authors and journals [17, 18], and author H‐index values were obtained from WoSCC.

## 3. Results

### 3.1. Overview of Publication Status

**FIGURE 1:**
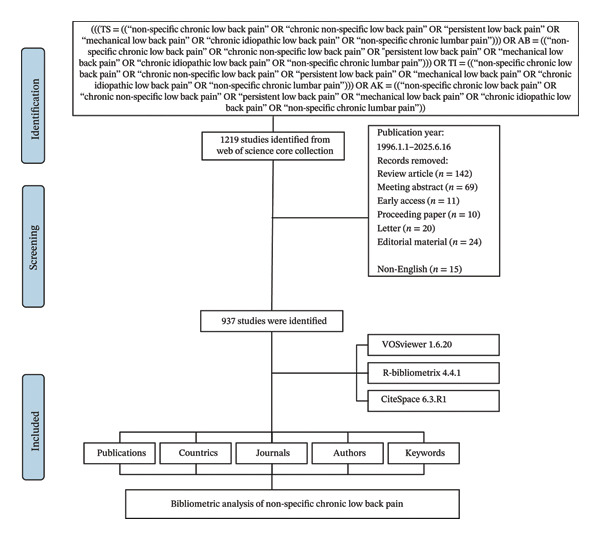
Flowchart of the literature screening process.

**FIGURE 2:**
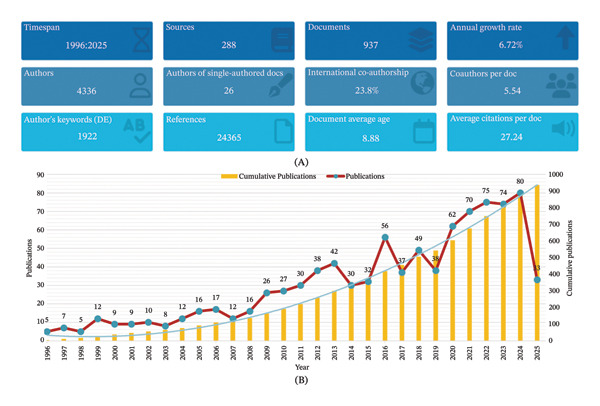
Publications overview from 1996 to 2025. (A) Collected studies overview; (B) Annual number of publications and trend.

A dataset of 937 eligible publications was compiled, as illustrated in Figure 1. These publications were authored by 4,336 individuals, published across 288 sources, and referenced a total of 24,365 works (Figure 2A). The publication trend reveals a consistent upward trajectory, with notable peaks in 2016 (56 publications), 2021 (75), and 2024 (80). Over the years, three distinct phases can be identified: the initial phase from 1996 to 2008, characterized by limited and sporadic output; a growth phase from 2009 to 2019, marked by steady increases and occasional fluctuations; and a rapid expansion phase from 2020 onward, during which annual publication output significantly accelerated. The apparent drop in 2025 is attributed to the dataset only covering the first half of the year (Figure 2B).

### 3.2. Analysis of Countries

**FIGURE 3:**
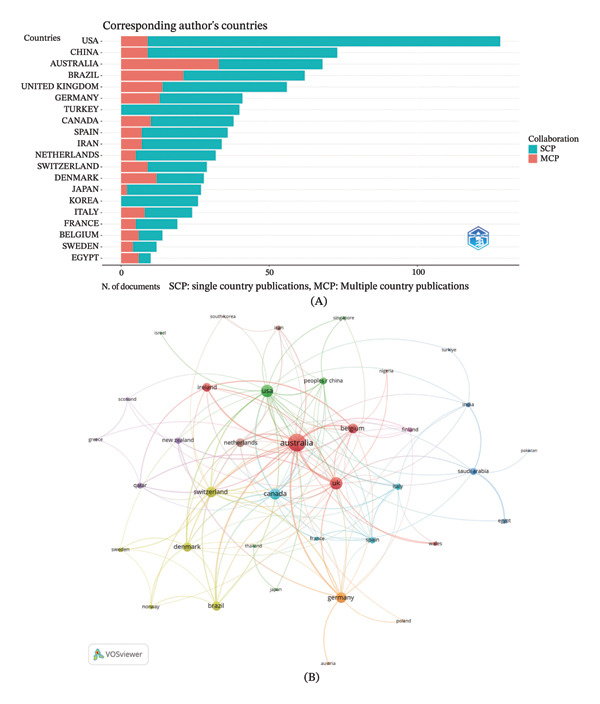
Analysis of countries. (A) Distribution of corresponding author’s publications by country. (B) Visualization map depicting the collaboration among different countries. Nodes represent countries, with size indicating publication count. Links represent coauthorships, with thickness showing collaboration strength. Colors indicate different research clusters. Total link strength in collaboration networks measures the frequency of coauthorship between countries, indicating the level of collaborative research. SCP: single‐country publications. MCP: multiple‐country publications.

The United States produced the largest number of publications (*n* = 128), followed by China (*n* = 73) and Australia (*n* = 68). For total publications (TPs), the highest value was also observed for the United States (*n* = 445), with Australia (*n* = 251) and China (*n* = 240) ranking next. For total citations (TCs), the United States took the lead (*n* = 5,532), followed by Australia (*n* = 2,754) and France (*n* = 2,604). Regarding average citations per publication, France ranked first (137.1), followed by the United States (43.2) and Australia (40.5) (Table 1). In terms of international collaboration, Australia exhibited a strong presence with a high proportion of multiple‐country publications (MCPs), accounting for 33 of 68 papers (MCP ratio = 0.485). Comparable levels of international collaboration were observed for Denmark and Belgium, with MCP ratios of 0.429 for each (Table 1 and Figure 3A). Among the 38 countries included in the collaboration analysis (minimum of five articles), the highest number of international collaboration links was identified for Australia (*n* = 128), followed by the United Kingdom (*n* = 65) and the United States (*n* = 65) (Figure 3B).

**TABLE 1:** Publication and citation profiles of leading countries.

Country	Articles	Freq	SCP	MCP	MCP_Ratio	TP	TP_rank	TC	TC_rank	Average citations
USA	128	0.137	119	9	0.070	445	1	5532	1	43.2
China	73	0.078	64	9	0.123	240	3	553	13	7.6
Australia	68	0.073	35	33	0.485	251	2	2754	2	40.5
Brazil	62	0.066	41	21	0.339	172	5	926	8	14.9
United Kingdom	56	0.060	42	14	0.250	176	4	1929	4	34.4
Germany	41	0.044	28	13	0.317	130	10	1190	5	29
Turkey	40	0.043	40	0	0.000	109	12	525	15	13.1
Canada	38	0.041	28	10	0.263	158	8	1068	6	28.1
Spain	36	0.038	29	7	0.194	162	7	822	9	22.8
Iran	34	0.036	27	7	0.206	105	13	457	17	13.4
Netherlands	32	0.034	27	5	0.156	164	6	1000	7	31.2
Switzerland	29	0.031	20	9	0.310	133	9	771	10	26.6
Denmark	28	0.030	16	12	0.429	119	11	650	11	23.2
Japan	27	0.029	25	2	0.074	72	17	542	14	20.1
South Korea	26	0.028	26	0	0.000	85	15	196	23	7.5
Italy	24	0.026	16	8	0.333	95	14	497	16	20.7
France	19	0.020	14	5	0.263	83	16	2604	3	137.1
Belgium	14	0.015	8	6	0.429	59	18	362	18	25.9
Sweden	12	0.013	8	4	0.333	35	20	236	21	19.7
Egypt	10	0.011	4	6	0.600	31	22	144	25	14.4

*Note:* Articles: publications of corresponding authors only. Freq: frequency of total publications. MCP_Ratio: proportion of multiple‐country publications. TP_rank: rank of total publications. TC_rank: rank of total citations. Average citations: The average number of citations per publication.

Abbreviations: MCP = multiple‐country publications, SCP = single‐country publications, TC = total citations, TP = total publications.

### 3.3. Analysis of Institutions

**FIGURE 4:**
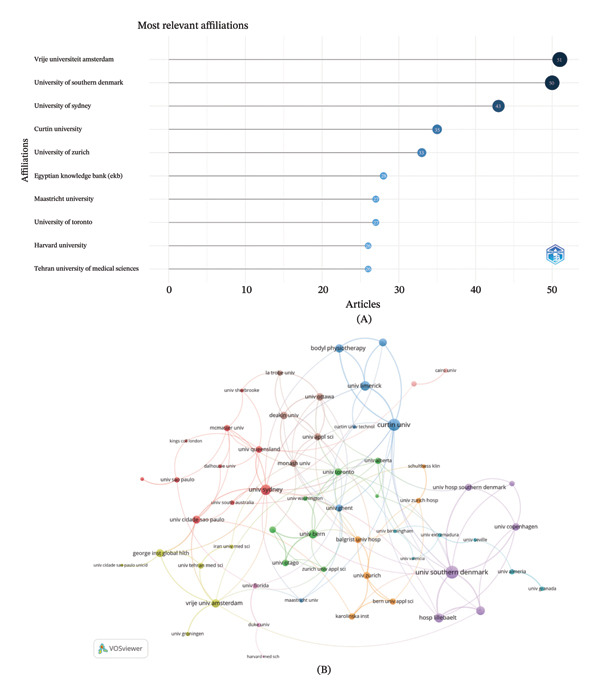
Analysis of institutions. (A) Top 10 institutions by article count and rank. (B) Institutions’ publications and collaborations: visual analysis. Nodes represent institutions, with size indicating publication count. Links represent coauthorships, with thickness showing collaboration strength. Colors indicate different research clusters. Total link strength in collaboration networks measures the frequency of coauthorship between institutions, indicating the level of collaborative research.

The top 10 institutions by publication output are shown in Figure 4A. Vrije Universiteit Amsterdam ranked first (51 publications), followed by the University of Southern Denmark (50) and the University of Sydney (43). Curtin University ranked fourth (35), and the University of Zurich ranked fifth (33). In the institutional collaboration analysis (65 institutions with at least five articles), the University of Southern Denmark showed the largest collaboration network (35 partnerships), followed by Curtin University (30) and the University of Sydney (23) (Figure 4B).

### 3.4. Analysis of Journals

The 20 journals publishing the most CNLBP‐related articles are summarized in Table 2. The highest output was observed for *BMC Musculoskeletal Disorders* (*n* = 53), followed by the *Journal of Back and Musculoskeletal Rehabilitation* (*n* = 41) and *European Spine Journal* (*n* = 38). Regarding TCs, *Spine* ranked first with 4,111 citations, followed by *Pain* (1,496) and *European Spine Journal* (1,132). The highest IF was observed for the *Journal of Physiotherapy* (9.7), despite its ranking of 24th by publication volume. Most of the leading journals were categorized as Q1 or Q2 in the JCR, with a few exceptions where IF data were unavailable (e.g., *Manual Therapy* and *Journal of Spinal Disorders and Techniques*). High H‐index values were also observed for *BMC Musculoskeletal Disorders* (21) and the *European Spine Journal* (18), respectively, demonstrating strong academic impact in the field.

**TABLE 2:** Bibliometric indicators of high‐impact journals.

Journal	H_index	G_index	M_index	IF 2023	JCR 2023	TP	TP_rank	TC	TC_rank	PY_start
BMC Musculoskeletal Disorders	21	33	0.875	2.2	Q3	53	1	575	7	2002
European Spine Journal	18	33	0.857	2.6	Q1	38	3	1132	3	2005
Manual Therapy	16	22	0.696	NA	NA	22	4	NA	NA	2003
Clinical Rehabilitation	15	21	0.750	2.6	Q1	21	6	156	37	2006
Spine	15	22	0.517	2.6	Q1	22	5	4111	1	1997
Journal of Back and Musculoskeletal Rehabilitation	12	18	0.545	1.4	Q3	41	2	208	24	2004
PLOS One	11	20	0.786	2.9	Q1	21	7	270	19	2012
Journal of Orthopaedic and Sports Physical Therapy	10	12	0.333	6	Q1	12	12	509	10	1996
Pain	10	11	0.345	5.9	Q1	11	16	1496	2	1997
Journal of Manipulative and Physiological Therapeutics	9	11	0.333	1.2	Q3	11	15	356	14	1999
Clinical Biomechanics	8	9	0.267	1.4	Q3	9	22	262	21	1996
European Journal of Physical and Rehabilitation Medicine	8	12	0.500	3.3	Q1	12	11	52	99	2010
Journal of Rehabilitation Medicine	8	12	0.421	2.5	Q2	12	13	113	46	2007
BMJ Open	7	12	0.636	2.4	Q1	17	8	99	50	2015
Brazilian Journal of Physical Therapy	7	10	0.438	3.1	Q1	10	19	79	60	2010
European Journal of Pain	7	7	0.538	3.5	Q2	7	31	470	11	2013
Journal of Physiotherapy	7	9	0.467	9.7	Q1	9	24	125	45	2011
Journal of Spinal Disorders and Techniques	7	10	0.292	NA	NA	10	20	NA	NA	2002
Physiotherapy	7	11	0.389	3.1	Q1	11	17	187	30	2008
Chiropractic and Manual Therapies	6	7	0.667	2	Q2	8	26	66	74	2017

*Note:* H_index: The H‐index of the journal, which measures both the productivity and citation impact of the publications. IF: The impact factor of the journal, indicating the average number of citations to recent articles published in the journal. JCR_Quartile: The quartile ranking of the journal in the JCR, indicating the journal’s ranking relative to others in the same field (Q1: top 25%, Q2: 25%–50%, Q3: 50%–75%, Q4: bottom 25%). PY_start: publication year start, indicating the year the journal started publication. TP: total publications. TP_rank: rank of total publications. TC_rank: rank of total citations.

Abbreviations: IF = impact factor, TC = total citations.

**FIGURE 5:**
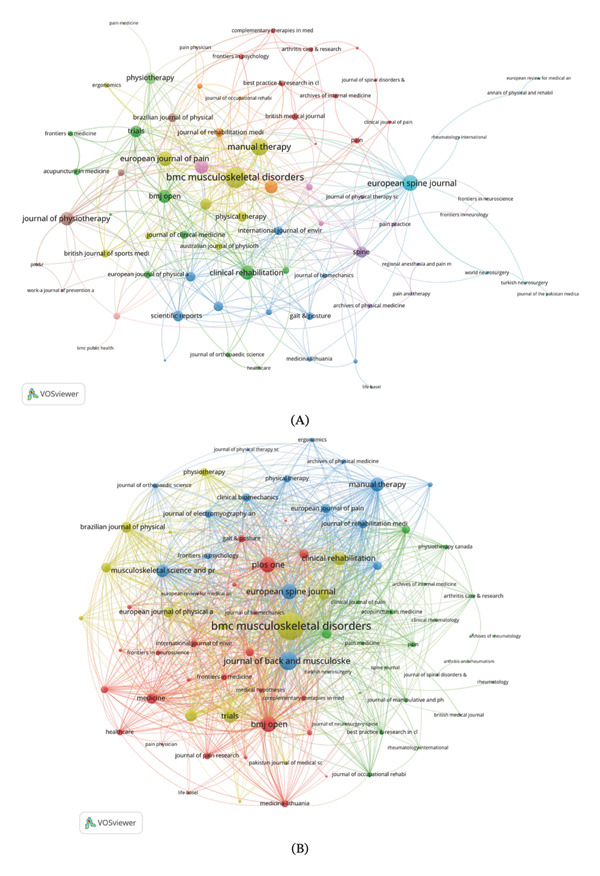
Analysis of journals. (A) Co‐occurrence network where node size = publication count and edge thickness = coappearance frequency; colors denote citation clusters identified by VOSviewer; (B) Coupling network illustrating shared reference patterns; total link strength quantifies similarity in reference lists.

The journal co‐occurrence network included 81 journals with at least three occurrences. The highest total link strength was observed for *BMC Musculoskeletal Disorders* (80), *Manual Therapy* (57), and *European Spine Journal* (49), as visualized in Figure 5A. The journal coupling network comprised 81 journals with at least three couplings, with the highest total link strength identified for *BMC Musculoskeletal Disorders* (13,524), *Journal of Back and Musculoskeletal Rehabilitation* (6,796), and *BMJ Open* (5,335) (Figure 5B).

### 3.5. Analysis of Authors

The 20 most productive authors in this field are listed in Table 3. Peter O’Sullivan leads in TPs with 18, followed by Kieran O’Sullivan with 13 and Chuhuai Wang with 9. The highest total citation counts were observed for Christopher G. Maher (834), followed by James H. McAuley (829) and Peter O’Sullivan (575). The highest H‐index was recorded for Peter O’Sullivan (14), followed by Kieran O’Sullivan (11); H‐index values of 6 were observed for several authors, including Wim Dankaerts, Chris G. Maher, and Anne Smith.

**TABLE 3:** Publication and citation profiles of high‐impact authors.

Author	H_index	G‐index	M‐index	PY_start	TP	TP_Frac	TP_rank	TC	TC_rank
O′sullivan Peter	14	18	0.93	2011	18	3.11	1	575	4
O′sullivan Kieran	11	13	0.73	2011	13	2.24	2	410	6
Bendebba M.	6	6	0.20	1996	6	1.29	9	210	15
Dankaerts Wim	6	7	0.40	2011	7	1.32	6	234	11
Maher Chris G.	6	7	0.33	2008	7	1.27	8	256	8
Smith Anne	6	6	0.50	2014	6	1.15	13	232	12
Geertzen Jan H. B.	5	6	0.25	2006	6	NA	NA	156	21
Leboeuf‐Yde Charlotte	5	5	0.28	2008	5	0.68	16	162	20
Long DM	5	5	0.17	1996	5	0.99	17	196	16
Maher Christopher G.	5	5	0.26	2007	5	0.88	18	834	1
Mataran‐Penarrocha Guillermo A.	5	5	0.36	2012	5	1.20	20	269	7
Mcauley James H.	5	5	0.26	2007	5	0.99	21	829	2
Wang Chuhuai	5	7	0.83	2020	9	1.06	5	62	42
Axen Iben	4	4	0.40	2016	4	0.88	26	196	16
Barz Thomas	4	4	0.40	2016	4	0.58	27	77	36
Breen Alan	4	4	0.31	2013	4	0.61	28	70	38
Fernandez‐Sanchez Manuel	4	4	0.50	2018	4	1.48	31	51	44
Glazov Gregory	4	4	0.29	2012	4	0.90	33	238	10
Hodges Paul W.	4	4	0.24	2009	4	0.62	34	154	22
Kent Peter	4	4	0.22	2008	4	0.58	15	120	26

*Note:* H_index: The H‐index of the author, measuring both productivity and citation impact of their publications. G_index: The G‐index of the author, giving more weight to highly cited articles. M_index: The M‐index of the author, calculated as the H‐index divided by the number of years since their first published paper. PY_start (publication year start): The year in which the author’s first publication appeared. TP (total publications): The total number of publications by the author. TP_rank (rank of total publications): The author’s rank based on the total number of publications. TC (total citations): The total number of citations received by the author’s publications. TC_rank (rank of total citations): The author’s rank based on the total number of citations. Average citations: The average number of citations per publication for the author.

**FIGURE 6:**
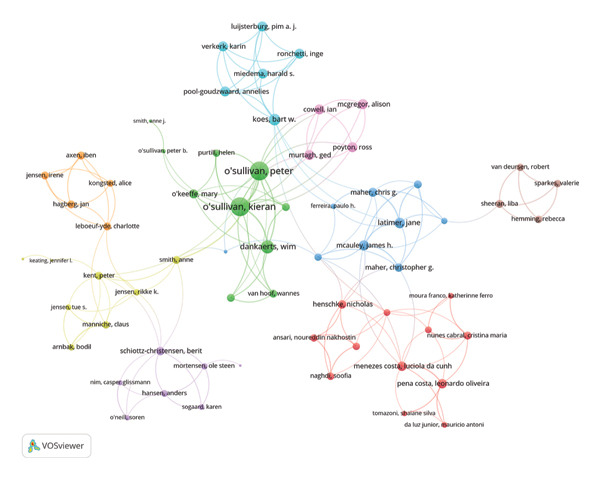
Author collaboration map produced in VOSviewer. Each node = an author (≥ 3 papers); size = number of publications; edge thickness = coauthorship frequency; colors = collaboration clusters (modularity class).

The author collaboration network was mapped in VOSviewer (Figure 6). Among the 175 authors meeting the inclusion criteria for collaboration analysis (at least three articles and international collaborations), the largest number of collaboration links was observed for Kieran O’Sullivan (46), followed by Peter O’Sullivan (45) and Wim Dankaerts (24).

### 3.6. Keywords Co‐Occurrence Analysis

**FIGURE 7:**
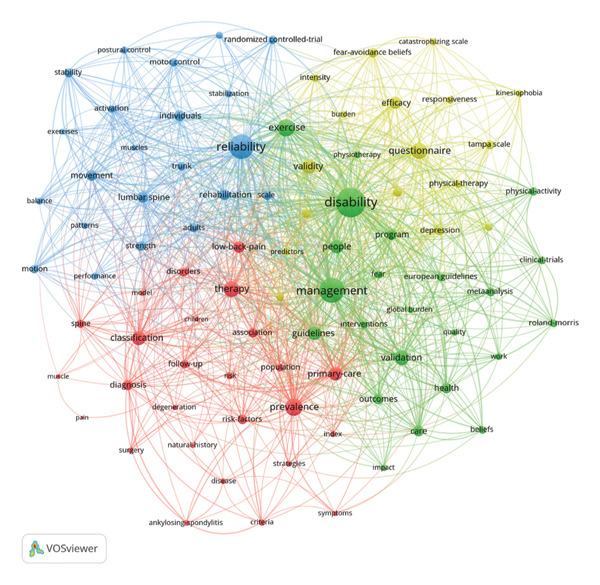
Visual analysis of keyword co‐occurrence network analysis. This network visualization displays the co‐occurrence of keywords in selected literature. Each node represents a keyword, with size indicating its frequency of occurrence. Links between nodes represent co‐occurrence in the same documents, with thicker lines showing stronger associations. Colors reflect the average publication year of the articles, as indicated by the color gradient at the bottom right.

Eighty‐eight high‐frequency keywords were identified and assigned to four thematic clusters (Figure 7). The red cluster (Cluster 1) primarily reflected disease phenotype and epidemiology, featuring terms like “classification,” “diagnosis,” “prevalence,” “risk factors,” and “population.” The green cluster (Cluster 2) was centered on evidence‐based policy and clinical management, including “guidelines,” “meta‐analysis,” “interventions,” “program,” and “physical activity.” The blue cluster (Cluster 3) emphasized neuromechanical function and rehabilitation techniques, with keywords such as “motor control,” “lumbar spine,” “postural control,” “stabilization,” and “exercises.” The yellow cluster (Cluster 4) highlighted psychosocial impact and patient outcomes, featuring terms like “fear‐avoidance beliefs,” “kinesiophobia,” “depression,” “quality‐of‐life,” and “questionnaire.”

### 3.7. Burst Keywords Analysis

**FIGURE 8:**
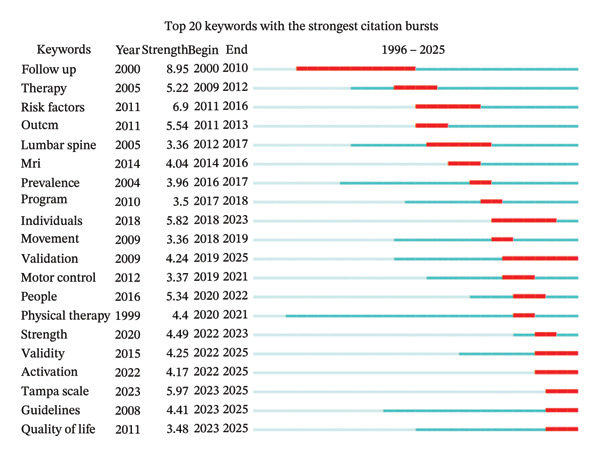
Top 20 keywords with the strongest citation bursts.

Analysis of the 20 keywords with the strongest citation bursts (2000–2025) indicated substantial shifts in research emphasis over time (Figure 8). The most pronounced bursts were identified for “follow up” (strength = 8.95, 2000–2010), “risk factors” (6.9, 2011–2016), and “tampa scale” (5.97, 2023–2025). In the early phase (2000–2010), research was oriented toward long‐term outcome tracking and treatment safety, as seen in keywords such as “follow up” and “therapy.” From 2011 to 2018, the focus began to shift toward epidemiological investigation and rehabilitation‐oriented classification. High‐burst terms like “risk factors,” “outcome,” “lumbar spine,” and “mri” suggest increased interest in identifying underlying contributors to chronicity and using imaging or structural data to inform treatment planning. During this period, methodological refinement gained momentum, as seen in “prevalence,” “program,” and “individuals.” Since 2019, the field has moved toward psychological assessment, validation studies, and individualized care models. Keywords such as “movement,” “motor control,” “people,” “physical therapy,” and “validation” all showed sustained citation bursts into 2025, pointing to a greater emphasis on patient function, precision rehabilitation, and measurement reliability. Notably, more recent bursts—such as “validity,” “activation,” “tampa scale,” “guidelines,” and “quality of life” (all starting from 2022 or later)—indicate rising interest in standardized outcomes, psychological dimensions of pain, and the quality‐of‐life effects of interventions for CNLBP.

## 4. Discussion

This bibliometric analysis reveals a clear and accelerating trajectory in CNLBP research over the past 3 decades. Beginning with limited early exploration, the field has experienced steady expansion since the mid‐2000s, followed by rapid growth after 2020. This shift corresponds with growing recognition of CNLBP as a multifactorial condition requiring biopsychosocial management and multidisciplinary collaboration. Thematic clusters identified in the co‐occurrence analysis—ranging from classification and diagnosis to psychosocial dimensions and rehabilitation—demonstrate the increasing complexity and clinical relevance of CNLBP research.

Australia has emerged as a pivotal contributor to CNLBP research, distinguished not only by its publication volume but also by the international reach and scientific influence of its output. Its leadership is underpinned by the prolific and highly cited output from institutions including the University of Sydney, Curtin University, and the University of Southern Denmark (via strong transnational partnerships). These academic centers have consistently driven forward evidence in movement‐based therapy, psychosocial modeling, and functional rehabilitation, forming the backbone of emerging clinical paradigms. Beyond sheer volume, Australian publications demonstrate a high average citation rate, reflecting strong academic influence and research quality. Seminal randomized controlled trials (RCTs) such as the RESOLVE trial [19] and motor skill training (MST) studies [20] have been published in high‐impact international journals such as *JAMA*, *JAMA Neurology*, and so on, further affirming Australia’s role in shaping global discourse on nonpharmacologic treatment for CNLBP. Moreover, Australia’s contributions are marked by a high degree of international collaboration, as evidenced by a substantial proportion of MCPs. Its coauthorship networks span Europe, North America, and Asia, facilitating multicenter trials and enhancing the cross‐cultural generalizability of findings. The prominence of Australian institutions in collaboration mapping (see Figure 3) further underscores their integrative role in global CNLBP research ecosystems. This combination of research impact and collaborative breadth positions Australia as a leading contributor and a key hub within the evolving CNLBP research landscape.

Notably, authors such as Peter O’Sullivan and Kieran O’Sullivan have been instrumental in advancing the understanding of CNLBP through their extensive publications. Peter O’Sullivan’s seminal classification review first framed CNLBP as a disorder of maladaptive movement and motor‐control impairment, laying the foundation for subgroup‐specific management (Man Ther 2005) [21]. He later issued an influential call to abandon purely biomedical models in favor of a biopsychosocial, patient‐centered approach to care (Br J Sports Med 2012) [22]. Building on this paradigm, Kieran O’Sullivan and colleagues demonstrated the practical effectiveness of cognitive functional therapy (CFT), a tailored intervention that integrates movement retraining with cognitive‐behavioral principles, in a multicase cohort of disabling CNLBP (Phys Ther 2015) [23]. Collectively, these contributions have redirected clinical guidelines toward holistic assessment and individually targeted rehabilitation strategies.

### 4.1. Thematic Structure and Temporal Evolution

#### 4.1.1. Four Distinct Research Clusters Were Identified Based on Keyword Co‐Occurrence Analysis

##### 4.1.1.1. Cluster 1: Epidemiological Patterns and Disease Definition

This cluster lays the groundwork for CNLBP research by addressing its definition, classification, and population‐level distribution. Keywords such as “classification,” “diagnosis,” “prevalence,” “risk factors,” and “population” reflect efforts to clarify clinical boundaries and assess societal burden. Relevant studies aim to improve diagnostic consistency and identify factors influencing disease onset and progression, providing the basis for cohort definition and preventive strategies. Variation in classification criteria has been noted in multiple sources. A scoping review protocol by Jess et al. pointed out inconsistencies in the timeframes used to define acute, subacute, and chronic low back pain across studies, with limited rationale for the specific durations adopted [24]. To address such inconsistencies, a formal taxonomy for chronic pain was proposed by the International Association for the Study of Pain (IASP) within the ICD‐11 framework, in which nonspecific chronic low back pain was classified as “chronic primary pain,” thereby reframing it from a symptom‐based construct to a disease category [25]. Epidemiological studies further characterize the affected populations. A cross‐sectional study of military personnel in Saudi Arabia reported a 2.7% prevalence of CNLBP and identified age, sleep quality, body mass index, smoking, and comorbidities as significantly associated with disability severity [26]. In a systematic review comparing subacute and chronic cases, Heitz et al. found that modifiable psychosocial risk factors were more frequently reported in the subacute phase, suggesting a temporal window where interventions may have greater preventive value [27]. Rodriguez et al. evaluated several classification schemes using the impact stratification score (ISS) and recommended an optimized system to improve patient grouping in epidemiologic and clinical research [28]. Building on this direction, Zheng et al. applied Mendelian randomization and confounder‐adjusted models to identify sleep disturbance, depression, and obesity as potential causal risk factors for CNLBP‐related disability [29], further refining the understanding of modifiable contributors at the population level.

##### 4.1.1.2. Cluster 2: Evidence‐Based Management and Health System Strategies

This cluster focuses on the formulation and dissemination of standardized, evidence‐based responses to CNLBP at both clinical and population levels. Keywords such as guidelines, meta‐analysis, interventions, management, and physical activity highlight the emphasis on systematic reviews, clinical consensus, and care frameworks aimed at improving treatment effectiveness and accessibility. Studies in this area underscore the global burden of CNLBP and advocate for cost‐effective, broadly applicable interventions, especially in primary care and resource‐limited settings. A comprehensive overview by Krenn et al. systematically analyzed recommendations from international guidelines and identified broad agreement on the use of nonpharmacologic interventions such as physical activity, though many recommendations were based on low‐ to moderate‐quality evidence [30]. Expanding on this, Nicol et al. critically reviewed international consensus statements, emphasizing the multimodal, stratified approach now endorsed across many countries [10]. To support effective treatment strategies, Abdelnaeem et al. conducted a systematic review of diagnostic classification systems, concluding that functionally oriented systems like O’Sullivan’s exhibit acceptable psychometric properties and may facilitate guideline implementation [31]. In parallel, the effectiveness of ultrasound therapy for CNLBP was evaluated by Haile et al., and its potential role as an adjunctive treatment within clinical practice frameworks was supported [32]. Meta‐analytic techniques are increasingly used to inform policy and clinical decision‐making. Zhang et al. performed a network meta‐analysis comparing acupuncture methods, identifying warm needle acupuncture and electroacupuncture as effective options for pain relief and mobility, information valuable for treatment recommendations in regions where acupuncture is accessible [33]. Moreover, in the context of global health equity, Motha et al. presented a protocol for a systematic review assessing physiotherapy self‐management programs in low‐ and middle‐income countries, highlighting the need for scalable, affordable strategies [34].

##### 4.1.1.3. Cluster 3: Neuromechanical Dysfunction and Rehabilitation Modalities

This cluster focuses on the neuromechanical underpinnings of CNLBP and their therapeutic implications. Central concepts such as motor control, lumbar stabilization, postural regulation, and neuromuscular adaptation define a body of research that bridges physical dysfunction with clinical rehabilitation. Interventions often target core musculature and sensorimotor feedback systems to restore trunk control and reduce pain. An RCT in university‐level musicians demonstrated that a 12‐week core stability exercise program significantly improved both pain intensity and functional status, highlighting the role of targeted trunk muscle conditioning in managing CNLBP [35]. Similarly, another study compared biofeedback sensor‐based training with conventional physiotherapist‐guided feedback during core stabilization exercises. Both methods produced equivalent improvements in disability, proprioception, and quality of life, supporting biofeedback as a viable tool for enhancing motor relearning [36]. Emerging neuroimaging evidence has further strengthened the link between neuromechanical interventions and central mechanisms. Two randomized trials of motor control exercise (MCE) reported not only symptom relief but also distinct patterns of brain reorganization. One found that MCE modulated effective connectivity between the default mode and frontoparietal networks, correlating with changes in pain perception and trunk muscle activation [37]. The other observed altered cerebellar–parietal connectivity, supporting MCE’s impact on sensorimotor integration and postural processing [38]. Beyond exercise, neuromodulatory approaches have also shown promise. Postural stability in patients with high pain‐related fear was significantly improved by transcranial direct current stimulation (tDCS) applied over the dorsolateral prefrontal cortex, indicating a top–down modulation of sensorimotor control [39]. In another trial, closed‐loop magnetic stimulation combining rTMS and peripheral stimulation reduced pain and activated somatosensory brain regions, suggesting synergistic effects on both peripheral and central pain pathways [40]. Collectively, these findings illustrate how neuromechanical dysfunction in CNLBP can be effectively addressed through targeted physical interventions, feedback mechanisms, and central neuromodulation, each contributing to measurable improvements in both symptomatology and functional capacity.

##### 4.1.1.4. Cluster 4: Psychosocial Dimensions and Patient‐Centered Outcomes

This cluster highlights the psychological and experiential aspects of CNLBP—fear‐avoidance beliefs, kinesiophobia, mood disturbance, and quality of life—that shape treatment response and long‐term prognosis. Recent cross‐sectional work in college athletes showed that higher scores on the Fear‐Avoidance Beliefs Questionnaire (physical‐activity subscale) were independently associated with the presence of CNLBP, underscoring avoidance behavior as a salient risk factor [41]. Complementary data from a Nigerian hospital cohort revealed that 92% of patients exhibited clinically relevant kinesiophobia, with self‐efficacy, pain intensity, and disability emerging as significant predictors [42]. Depression and somatization also remain pivotal: a large U.S. trial found both constructs strongly correlated with pain severity, back‐specific disability, and poorer general health, reinforcing their role in the biopsychosocial profile of CNLBP [43]. Robust measurement tools are essential for personalized care. The Persian Brief Illness Perception Questionnaire demonstrated excellent reliability (Cronbach’s *α* = 0.90; ICC = 0.90) and a clear two‐factor structure, providing a validated instrument for assessing illness perceptions in Persian‐speaking CNLBP populations [44]. Intervention studies further illustrate how psychosocial targets translate into outcomes: a single session of Kinect‐based exergaming produced larger immediate reductions in pain, fear‐avoidance beliefs, and negative mood than core‐stability exercises, suggesting that engaging digital modalities can modulate cognitive–affective factors alongside symptoms [45]. Finally, in a randomized trial comparing mindfulness‐based stress reduction with cognitive‐behavioral therapy, baseline mindfulness “non‐judging” scores moderated functional and pain improvements, while lower anxiety and stronger pain‐control beliefs predicted benefit regardless of treatment arm [46]. Collectively, these studies demonstrate that psychosocial constructs are both measurable and modifiable, supporting multidimensional assessment and tailored interventions as cornerstones of patient‐centered CNLBP management.

An analysis of citation bursts reveals a structured, time‐dependent evolution in CNLBP research priorities from 2000 to 2025. Three partially overlapping phases can be distinguished‐disease characterization, mechanistic refinement, and biopsychosocial validation‐each marked by high‐intensity burst keywords that illuminate the field’s shifting focus.

The first phase is dominated by attempts to delineate what CNLBP is and how it evolves over time. The most prominent burst—“follow up” (strength = 8.95, 2000–2010)—captures a wave of foundational studies that tracked symptom persistence and functional outcomes. Related bursts such as “therapy” (2009–2012) and “prevalence” (2004–2017) reflect the growing use of population surveys and exploratory clinical trials aimed at understanding CNLBP’s burden and treatment potential. For instance, a large cross‐sectional study of Tunisian adolescents reported a lifetime prevalence of 28.4% and identified risk factors such as school dissatisfaction and family history [47]. In parallel, Williams et al. tested the efficacy of Iyengar yoga in a randomized trial, finding significant improvements in pain intensity, disability, and medication use, suggesting promise for complementary interventions even in mild chronic cases [48]. At the same time, diagnostic clarity remained limited. Petersen et al. evaluated a novel pathoanatomic classification system for CNLBP and found moderate intertester reliability (*κ* = 0.62), reinforcing the challenge of consistent clinical assessment in heterogeneous patient populations [49]. Together, these early efforts formed a necessary foundation for future refinement of definitions, outcomes, and subgroup‐specific treatment strategies.

Between 2011 and 2018, the spotlight shifted toward why pain persists. Citation bursts for “risk factors” (2011–2016), “lumbar spine” (2012–2017), and “mri” (2014–2016) mark a surge of imaging‐based and pathoanatomical work. A prospective study using hybrid SPECT‐CT showed that metabolically “hot” spinal segments were more than twice as likely to appear in patients with CNLBP than in controls, suggesting occult inflammatory or degenerative generators that routine MRI may miss [50]. Complementing these findings, a cross‐sectional analysis linked Modic changes on MRI to both hypovitaminosis D and obesity, reinforcing metabolic load as a modifiable contributor to vertebral end‐plate pathology [51]. Concurrent bursts for “program” (2017–2018) and “individuals” (2018–2023) reflect the rise of stratified rehabilitation. Field studies began to identify occupation‐specific subgroups: among Thai rice farmers, clinical lumbar instability was twice as prevalent in those with ≥ 30 years of farming, pointing to the need for tailored core‐stability programs rather than generic exercise prescriptions [52]. Collectively, these investigations moved the field from descriptive epidemiology to mechanism‐oriented imaging and subgroup‐specific intervention planning, setting the stage for later consensus guidelines that advocate multimodal, individualized care pathways.

Since 2019, research has converged on validating how integrated interventions influence function and quality of life. A growing body of RCTs has tested neuromuscular re‐education and sensorimotor retraining approaches, reflecting thematic bursts in “movement” (2019–2025), “motor control” (2021–2024), and “strength” (2022–2023). For instance, MST demonstrated greater long‐term functional improvement compared to traditional strength/flexibility protocols [20], while graded sensorimotor retraining (RESOLVE) significantly reduced pain intensity at 18 weeks [19]. DNS‐based protocols further enhanced functional movement and balance, especially in older adults [53]. Parallel emphasis on measurement integrity is evident from sustained bursts in “validation” (2019–2025) and “validity” (2022–2025), supporting refinement of outcome tools. The Persian versions of the FABQ, TSK, and PCS demonstrated high responsiveness and defined MIC thresholds, facilitating their application in clinical trials [54]. Psychosocial constructs have risen to prominence, with the “Tampa Scale” (2023–2025) underscoring fear‐avoidance assessment. Recent modeling confirmed that pain catastrophizing precedes fear of movement, which in turn predicts disability and pain [55]. This supports the cognitive‐behavioral framework underlying several modern interventions. Quality of life (2023–2025) has become a central outcome. A pilot RCT involving behavioral therapy via VR showed significant improvement in daily pain experience, although not in SF‐12 QoL metrics [56]. Meanwhile, evidence suggests that simple self‐stretching protocols are comparable to motor control programs, emphasizing the value of patient‐preferred, cost‐effective interventions [57].

Looking ahead: Persistent bursts for guidelines, activation, and psychosocial metrics through 2025 suggest continued interest in precision rehabilitation, digital activation monitoring, and whole‐person outcomes. Future research is likely to integrate wearable sensor data, machine‐learning risk prediction, and cost‐effectiveness analyses, driving individualized CNLBP care, especially in primary care and aging populations.

### 4.2. Strengths and Limitations

A comprehensive bibliometric analysis of the global CNLBP research landscape over the past 3 decades was conducted. Using a large set of peer‐reviewed articles retrieved from the WoSCC, the development of the field was characterized, and key contributors, collaboration networks, and thematic patterns were identified. Robustness was strengthened through the combined use of established tools (bibliometrix, VOSviewer, and CiteSpace), enabling cross‐checking of findings across platforms. Relative to narrative or systematic reviews, bibliometric methods allow a broader and more quantitative mapping of research hotspots and underlying knowledge structures. It effectively uncovers the evolution of scholarly focus—from early epidemiological classification efforts to recent emphasis on personalized, evidence‐based rehabilitation strategies—and highlights emerging keywords and clusters that signal the future direction of CNLBP research.

Despite these strengths, there are several limitations in this study. First, restriction to English‐language records indexed in a single database may have introduced language and selection biases and may have excluded relevant work from non‐English‐speaking regions. Second, the exclusive focus on peer‐reviewed journal articles resulted in the omission of gray literature, including conference proceedings, theses, and policy reports, which may provide early signals of emerging topics or perspectives that are less represented in journal publications. Third, although citation‐based indicators are widely applied, research quality and clinical relevance may not be fully reflected, particularly for recent publications that have had limited time to accumulate citations. Furthermore, although the analysis provides a high‐level overview of trends and patterns, it does not involve a critical appraisal of individual study methodologies, results, or real‐world implications—an aspect that would require systematic review approaches. Finally, as with all bibliometric studies, the data represent a snapshot in time; given the fast‐paced evolution of research—particularly in domains such as digital therapeutics, machine learning, and behavioral science—new themes may emerge that have not yet registered as citation bursts.

In conclusion, valuable insights into the historical trajectory and current priorities of CNLBP research were provided by this study. It confirms a shift from general classification frameworks toward more targeted, interdisciplinary approaches focused on function, patient experience, and long‐term outcomes. Future research should aim to address the identified gaps through the integration of qualitative assessments, incorporating diverse data sources, and emphasizing clinical translation. As the field continues to evolve, especially with advancements in digital health and personalized medicine, bibliometric analyses will remain essential tools for guiding evidence‐based innovation and strategic research planning.

## Author Contributions

Wei Yan, Xiaobing Xi, and Min Fang designed the study, collected, reviewed, analyzed data, and wrote the manuscript. Lingjun Kong, Tianxiang He, Xin Zhou, and Guangxin Guo verified data and contributed to data analysis. Qingguang Zhu, Xiaobing Xi, and Min Fang revised the manuscript and provided valuable suggestions for study design and data analysis. Qingguang Zhu, Xiaobing Xi, and Min Fang contributed equally, designed the project, edited the manuscript, and supervised the study.

## Funding

This study was supported by the Traditional Chinese Medicine Research Project of Shanghai Municipal Health Commission (2024QN110), Shanghai Key Laboratory of Traditional Chinese Medicine Tuina Technology for Muscle and Bone Diseases (24dz2260200), Three Year Action Plan Project for Shanghai to Further Accelerate the Inheritance, Innovation and Development of Traditional Chinese Medicine (ZY (2025–2027)–3–1–1), Construction of research‐oriented wards of SHDC (SHDC2022CRW010), National Natural Science Foundation of China (82205304).

## Disclosure

All authors have approved the final version of this paper.

## Ethics Statement

The authors have nothing to report.

## Consent

The authors have nothing to report.

## Conflicts of Interest

The authors declare no conflicts of interest.

## Data Availability

All data generated or analyzed during this study are included in this article.
